# Ocular Symptoms as an Aid in General Diagnosis

**Published:** 1909-03-13

**Authors:** William Anderson

**Affiliations:** Ophthalmic Assistant Registrar and Tutor, Guy's Hospital; late Senior House Surgeon, The Royal London Ophthalmic (Moorfields) Hospital; Medical Officer, Metropolitan Asylums Board Ophthalmic Schools, Swanley


### Hospital Clinics.

### OCULAR SYMPTOMS AS AN AID IN GENERAL DIAGNOSIS.

By WILLIAM ANDERSON, M.A., M.B., B.Ch.; Ophthalmic Assistant Registrar and Tutor, Guy's Hospital; late Senior House Surgeon, The Royal London Ophthalmic (Moorfields) Hospital; Medical Officer, Metropolitan Asylums Board Ophthalmic Schools, Swanley.

The endeavour to obtain accurate diagnosis by the collection of objective evidence of disease is a happy feature of modern medicine. Many ocular symptoms are objective and easily observed, others are subjective and readily ascertained. Some of these symptoms afford the earliest indications of a threatened general disease, others reliable evidence of past disease, or they may be a valuable aid in differential diagnosis. I propose to discuss the symptoms presented by the different parts of the eye in order, beginning with the lids.

One morning a man came to Moorfields "because his lids drooped." This had begun gradually fourteen years previously, in each eye. After a few attacks of influenza the drooping increased more rapidly, so that his vision was considerably impaired. At first it appeared to be a case of ptosis, which is of two kinds, congenital or paralytic. The history is not consistent with either, and, moreover, he was able to raise his lids, though unable to maintain them in this position. Repetition of this movement soon produced exhaustion, and he was unable to raise the lids again till after a period of rest.

An examination of the other muscles of the body showed that they all, except the sphincters, were markedly weak; and the patient was in fact suffering from myasthenia gravis. Excluding the two varieties of ptosis and the drooping of the lids, associated with old trachoma, a similar condition is an early symptom of this disease.

Prominence of the eyeballs is perhaps the most striking feature of Graves' disease. This is accentuated, or it may be only simulated, by retraction of the upper lid—an infrequency of the winking movements, known as "Stellwag's sign." The familiar staring facial expression thus produced suggests at once an examination for further clinical evidence. Ask your patient to look down. If the upper lid lags behind the eyeball in its descent, so that a large part of the sclerotic is exposed, we have "von Græfe's sign," of no little importance, for though it is not always present, it does not occur in other kinds of exophthalmos, and, on the other hand, it is sometimes observed before the protrusion of the eyeballs. The enlargement of the thyroid, tachycardia, and muscular tremors complete the diagnosis. The opposite condition, recession of the eyeball, with slight drooping of the upper lid (muscle of Müller), is present in paralysis of the cervical sympathetic nerve.

Abnormal and defective movements of the eye are easily observed. Involuntary clonic contractions of the muscles of the eyeball, usually symmetrical, equal in both eyes, lateral, vertical or rotary in direction, not infrequently occur. Such nystagmus is most commonly found with congenitally defective vision—microphthalmos, disease of the choroid, and in albinos. But when it is associated with normal eyes and of recent origin, it is a valuable indication of the probable presence of disseminated sclerosis; and it is the only eye symptom which occurs with any constancy in Friedreich's disease (hereditary ataxia).

Loss of power of one or more muscles of the eyeball is always to be regarded as a symptom, not as itself a disease. The characteristic symptom of paralysis of the orbital muscles is diplopia, and by its means we ascertain the muscle or muscles involved. The lesions causing ocular paralysis may affect the nerve trunks, as syphilitic and rheumatic inflammations, presence of new growths, orbital or intracranial, or of aneurysm. Again, the lesion may be in the nerve nuclei, or in the fibres connecting the nerves with their nuclei, such as hæmorrhage, softening, tumours, and disseminated sclerosis. It is important to determine the site of the lesion. Apart from injury and orbital disease, isolated paralysis is most often due to a basal lesion. If the internal muscles are alone paralysed in both eyes, or the external muscles alone, or if associated muscles in the two eyes are paralysed at the same time, producing conjugate deviation, the lesion must be nuclear, or, in the case of conjugate deviation, supra-nuclear.

The localising value of paralysis of the ocular muscles in cerebral disease is of considerable importance, though we must not forget that the paresis may be a distant symptom. This is especially so with the sixth nerve, for its lengthened course over the most prominent part of the pons renders it readily affected by distant pressure.

Paralysis of the orbital muscles occurs in about 30 per cent. of the cases of locomotor ataxy, usually in the pre-ataxic stage or even as an early symptom. It is of two kinds—a transient paralysis, which lasts a few days or weeks and may recur, or a permanent paralysis of one or two muscles. This may be of value in directing our examination for other more definite symptoms of the disease. Again, especially in children, paralysis of ocular nerves may follow an attack of diphtheria, and in that case other evidence of post-diphtherial nerve poisoning must be looked for.

In the cornea, interstitial keratitis affords us valuable evidence of inherited syphilis. An attack of iritis may be due to syphilis, rheumatism, tuberculosis, or diabetes, and so we must look for other symptoms which the iritis suggests to our mind.

Abnormalities of the pupil and accommodation are of considerable diagnostic importance. Marked inequality in the size of the pupils is produced by an irritation of the cervical sympathetic, which may be caused by the pressure of a tumour or carotid aneurysm. The pupils are often unequal in general paralysis of the insane and sometimes contracted.

You are all familiar with the Argyll Robertson pupil, which reacts to accommodation but not to light; this is most characteristic of locomotor ataxy, and often early evidence of its onset. It is occasionally met with in general paralysis. Dilated pupils are seen in epilepsy, and sometimes abnormal oscillations, called "hippus."

Paralysis of the accommodation is a not infrequent sequela of diphtheria. The pupils react to light and are not usually dilated; the distant vision may be quite good, though the patient is unable to read. The condition lasts a few weeks and is always an indication to be on the outlook for graver sequelæ.

Cataract may be associated with diabetes. I recall the case of a woman who was admitted to Moorfields about four years ago for extraction of cataract. She was apparently in good health, but an examination of the urine showed the presence of a considerable amount of sugar. The operation was postponed and the patient was dieted. Three days after admission coma came on, and death took place on the same evening. It is very important to examine the urine in all cases of cataract.

We come now to those parts of the eye, to examine which we must use the ophthalmoscope. By means of this instrument we can see certain parts of the nervous and vascular systems and note changes in them, which may mean some generalised pathological condition or disease localised within the skull or in some more remote part of the body.

Pathological conditions may be present in the fundus without any defect of vision. Many years ago Hughlings Jackson pointed out that in even severe optic neuritis the vision may be normal. Probably the first suggestion that occurs to the physician when a double optic neuritis is present is the diagnosis of intra-cranial tumour. Double optic neuritis may be seen in other conditions, for instance, chlorosis and lead-poisoning. When, however, it is associated with severe headache, vomiting or other cerebral symptom, the diagnosis is practically certain. Usually optic neuritis is a comparatively late result of intra-cranial tumour, but there are exceptions to the rule, and it may be the first and for some time the only symptom of such a tumour.

When a patient complains of occasional headache, with or without vomiting and no defect of sight, it is a wise precaution to make an ophthalmoscopic examination.

In another class of cases there is, in addition, some dimness of vision, and the optic disc presents an appearance closely simulating that found in brain tumour. I mean where an error of refraction—hypermetropia—is the cause. The chief differences between this pseudo- and tumour-papillitis are in the condition of the physiological pit and of the vena centralis retinæ; in the former the pit is not obliterated and the vein not engorged.

Convulsions are not so frequent in brain tumour as the symptoms already mentioned. Sometimes they occur, general and epileptiform in character, and so a diagnosis of idiopathic epilepsy may be made. This is further confirmed for a time by an improved condition under a course of bromides, till the discovery of double optic neuritis reveals the mistake. Optic neuritis is of little value in estimating the size, nature, or localisation of the new growth, but it is of some value in deciding if surgical interference is likely to preserve sight.

A very different appearance of the disc—white with contracted vessels—is seen in optic atrophy. This occurs in about 10 per cent. of the cases of locomotor ataxy, and commences more frequently in the pre-ataxic period than subsequently. Sometimes it is the first symptom of the disease, and its diagnostic value is appreciated most in those cases where the spinal symptoms are not marked. Thus a patient comes complaining of a certain difficulty in emptying the bladder or some pains in the lower limbs. These may mean one thing or another, but if there are signs of optic atrophy or the knee-jerks are absent, the significance of the combined symptoms is evident. So, too, the ophthalmoscope may reveal to us that repeated attacks of gastric disorder are in reality gastric crises.

Albuminuric retinitis is one of the most familiar retinal changes of considerable importance in medical practice.

It is perhaps of more value in prognosis, for when it is present in renal disease there are usually other evidences of this affection, and the retinal complication means that the end of life is drawing near. The only exception to this grave prognosis is when the inflammation is acute, as in post-scarlatinal nephritis, or in the albuminuria of pregnancy. Its diagnostic value is greater in exceptional cases, where kidney disease exists without albuminuria, and it is of some value in distinguishing organic from functional albuminuria. Further, it is the ophthalmic surgeon who is, not infrequently, the first to discover the renal lesion; the patient may feel in good health, but some interference with the vision leads to an ophthalmoscopic examination, and so to the discovery of the retinal disease and to subsequent examination of the urine. Retinal changes not unlike those seen in albuminuric retinitis occur in diabetes. The ultimate diagnosis depends on the presence of albumen or sugar in the urine.

In the retina we have the unique privilege of seeing the minute arteries. Degeneration of their walls—the so-called "silver wire" arteries—is often the first observation by which we are able to diagnose granular kidney disease, and at a later stage to anticipate the imminence of cerebral vascular disease.

It is often difficult to obtain a history of syphilis from a patient, but lesions of the eye afford us valuable evidence of its activity, and this aid in securing a diagnosis is of the first importance. Thus in the fundus oculi there may be patches of choroidal atrophy, or a diffuse retinitis with opacities in the vitreous. I have already referred to attacks of iritis, and we may find evidence of former attacks in the presence of posterior synechiæ and pigment deposits on the anterior capsule of the lens.

You know well how difficult it is at times to distinguish between organic and functional disorder. Disseminated sclerosis in its early stages is most commonly mistaken for hysteria, and here the eye symptoms may come to our aid:—

| | Disseminated Sclerosis | Hysteria |
|---|---|---|
| Oculo-motor disturbances | Nystagmus and isolated paralysis of orbital muscles | Rarely occur |
| Fundus ... ... | Optic atrophy | Normal |
| Field of vision ... | Central colour scotoma | If deranged, is contracted |

Failure of vision is common enough in hysteria, but optic atrophy obviously means something more than functional disturbance.

The intra-ocular tension should not escape your observation in some conditions. Acute glaucoma is often accompanied by violent headache, severe vomiting, and rise of temperature, and so it is not infrequently mistaken for influenza or some gastric trouble. An examination of the eyes clears up the diagnosis.

A subjective symptom of considerable importance is hemianopia. It is evidence of intra-cranial disease, the site of the lesion being in one optic tract, the visual centre in one occipital lobe, or in the fibres connecting the tract and centre.

The patient complains of being blind in one eye, when in reality he is blind to objects on that side—*e.g.* if the lesion is in the right optic tract, this produces loss of function in the temporal half of the retina on the right side and in the nasal half of the retina on the left side, so that there is only half vision, and the patient is blind to objects on the left side. This is termed homonymous hemianopia, and the lesion may be in (1) the optic tract between the chiasma and the lateral geniculate body; (2) in the central fibres between the geniculate body and the cerebral cortex; (3) in the cuneus (occipital lobe).

Whether the lesion is in the tract in front of the geniculate bodies or behind in the central portion can be determined by Wernicke's hemiopic pupil. Light is thrown into the eye so that it falls on the blind half of the retina. If the pupil contracts the lesion must be beyond the geniculate body—*i.e.* behind the pupil centre. If the pupil remains inactive the lesion is in the optic tract.

When the lesion is in the central portion of the optic chiasma the nasal halves of the retinæ are involved, producing loss of vision in the outer half of each field, or what is known as bitemporal hemianopia. This is found in tumours or hypertrophy of the pituitary body, and may be an early symptom of acromegaly.

